# A constitutive active MAPK/ERK pathway due to BRAF^V600E^ positively regulates AHR pathway in PTC

**DOI:** 10.18632/oncotarget.5194

**Published:** 2015-09-16

**Authors:** Gianluca Occhi, Susi Barollo, Daniela Regazzo, Loris Bertazza, Francesca Galuppini, Vincenza Guzzardo, Marie Lise Jaffrain-Rea, Federica Vianello, Denis Ciato, Filippo Ceccato, Sara Watutantrige-Fernando, Andrea Bisognin, Stefania Bortoluzzi, Gianmaria Pennelli, Marco Boscaro, Carla Scaroni, Caterina Mian

**Affiliations:** ^1^ Department of Biology, University of Padova, Padova, Italy; ^2^ Endocrinology Division, Department of Medicine, Hospital/University of Padova, Padova, Italy; ^3^ Surgical Pathology & Cytopathology Unit, Department of Medicine, Hospital/University of Padova, Padova, Italy; ^4^ Department of Clinical and Biotechnological Sciences, University of L'Aquila, L'Aquila, Italy; ^5^ Neuromed Institute, Department of Neurological Sciences, University of L'Aquila, L'Aquila, Italy; ^6^ Department of Radiotherapy, Istituto Oncologico del Veneto, IOV-IRCCS, Padova, Italy; ^7^ Department of Molecular Medicine, University of Padova, Padova, Italy

**Keywords:** papillary thyroid cancer, aryl hydrocarbon receptor, BRAF, gene expression, meta-analysis

## Abstract

The aryl hydrocarbon receptor (AHR) is a ligand-activated transcription factor mediating the toxicity and tumor-promoting properties of dioxin. AHR has been reported to be overexpressed and constitutively active in a variety of solid tumors, but few data are currently available concerning its role in thyroid cancer. In this study we quantitatively explored a series of 51 paired-normal and papillary thyroid carcinoma (PTC) tissues for AHR-related genes. We identified an increased AHR expression/activity in PTC, independently from its nuclear dimerization partner and repressor but strictly related to a constitutive active MAPK/ERK pathway. The AHR up-regulation followed by an increased expression of AHR target genes was confirmed by a meta-analysis of published microarray data, suggesting a ligand-independent active AHR pathway in PTC. *In-vitro* studies using a PTC-derived cell line (BCPAP) and HEK293 cells showed that BRAF^V600E^ may directly modulate AHR localization, induce AHR expression and activity in an exogenous ligand-independent manner. The AHR pathway might represent a potential novel therapeutic target for PTC in the clinical practice.

## INTRODUCTION

Thyroid cancer is considered the most common endocrine malignancy and the incidence of papillary thyroid carcinoma (PTC), its most frequent histologic subtype - representing approximately 80% of all thyroid cancers - [1], is rapidly increasing [2].

About 36%-69% of PTCs harbor activating mutations in the *BRAF* gene (v-raf murine sarcoma viral oncogene homolog B1), which encodes a member of the raf/mil family of serine/threonine kinases. BRAF functions to regulate the MAPK/ERK pathway transducing extracellular stimuli to the nucleus [3]. Active BRAF phosphorylates and activates MEK1/2 beginning a kinase cascade that, through ERK1/2, signals for ligand- and cell-specific responses. More than 90% of the BRAF mutated PTCs are characterized by a T1799A transversion that results in the V600E aminoacidic substitution (BRAF^V600E^) within the activating domain of the protein [4]. Disrupting hydrophobic interactions, BRAF^V600E^ enables the protein to fold into a catalytically active form with a nearly 500-fold increased kinase activity [5]. This event leads to the activation of the downstream signaling cascade in the absence of extracellular stimuli, allowing the cell to become self-sufficient in growth signals within this pathway [6]. In transgenic mice, BRAF^V600E^ induces the development of thyroid cancer with high penetrance and short latency, thus suggesting that BRAF mutations may function as the initial transforming event during thyroid tumor development [7]. All these data support the central role of BRAF^V600E^ and MAPK signaling pathway in transformation in PTC, however the mechanism of concomitant activation of different signaling pathways by BRAF^V600E^ and their effects in thyroid cancer are not fully elucidated.

Aryl hydrocarbon receptor (AHR) is a ligand-activated transcription factor that mediates the effects of many environmental pollutants, including polycyclic aromatic hydrocarbons (PAH) and 2,3,7,8-Tetrachlorodibenzo-*p*-dioxin (TCDD), through the induction of several phase I (e.g. CYP1A1, CYP1B1) and II biotransforming enzymes (e.g. UDP-glucoronosyl transferase UGT1A6, NADPH-quinone-oxidoreductase, NQO1) [8]. In its ligand-free inactive form, the AHR is a cytosolic protein complexed to two HSP90 molecules, the HSP90-interacting protein p23 and the AHR-interacting protein AIP [9]. Upon ligand binding, AHR translocates to the nucleus where it heterodimerizes with the aryl hydrocarbon nuclear translocator (ARNT). This active complex binds to xenobiotic-response elements (XRE) located in the enhancer/promoter regions, thus regulating the expression of target genes [8].

AHR has long been studied for its role in mediating TCDD toxicity [10]. However, its involvement in many other biological processes, including development, immunity and cancer biology, is strongly emerging [8, 11, 12]. AHR is overexpressed and constitutively active in a variety of tumor types, in cancer cell lines and in tumors from animal models, where it mostly shows a pro-oncogenic role [12]. A recent study on multiple cell lines from the Cancer Cell Line Encyclopedia reported relative high level of AHR mRNA in some cellular models of solid tumor (e.g. pancreatic, liver and chondrosarcoma derived cell lines); low levels were instead detected in many leukemia subtypes [13]. More recently, investigating the role of genetic events responsible for the onset of the thyroid cancer in acromegaly, we have shown that AHR is selectively overexpressed in a small cohort of PTC compared to the normal surrounding tissue. We found, in addition, that such increase was more marked in PTC samples harboring BRAF^V600E^, irrespective of acromegaly status [14]. Given the proven cross-talk between AHR and MAPK pathway [15] and the role of AHR in modulating growth and migration of cancer cells [12], in the present work we aim to confirm previous associations between the BRAF^V600E^ and AHR overexpression in a large, independent cohort of patients with PTC. In addition, cellular models of PTC are used to systematically analyze the expression levels of other components of the AHR signaling pathway, and to better elucidate the molecular link between BRAF^V600E^ and AHR in thyroid tumor.

## RESULTS

### AHR, AHRR and ARNT expression in tumor and paired normal tissue

**Figure 1:**
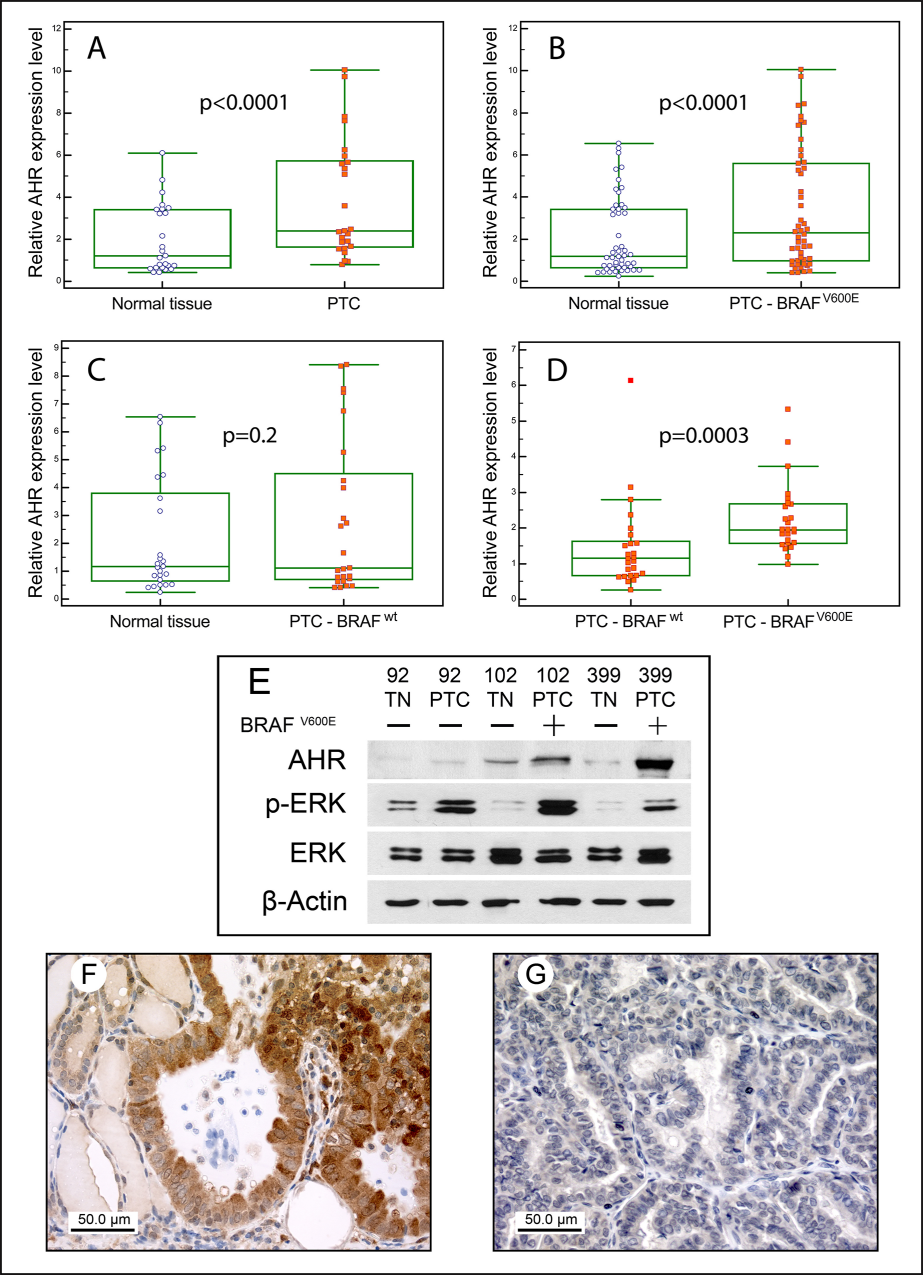
AHR expression in PTC Box plots of relative qPCR gene expression measurements of *AHR* in either all PTCs **A.** only the BRAF^V600E^ **B.** or BRAF^wt^ **C.** PTCs and the relative paired normal tissues. Each value was referred to a pool of normal thyroid tissues that was set to 1. In **D.** the *AHR* expression of BRAF^V600E^ *vs* BRAF^wt^ PTCs is shown. For each sample the reported value represents the fold increase in tumoral specimen compared to its normal counterpart, which was set to 1. Boxes indicate the range from lower to upper quartile values, with the line inside the box representing the median. The vertical lines mark the highest and lowest value observed within a distance of 1.5 times the inter-quartile range from the bottom and the top of the boxes, respectively. Each dot represents a single sample. **E.** Representative Western blot analysis of normal/tumoral match-pair samples for the expression of AHR, phospho-ERK and total ERK. Samples were corrected for protein loading by β-Actin and the BRAF mutational status was reported on the top. AHR immunostaining in BRAF^V600E^ **F.** and in BRAF^wt^ **G.** PTC samples. Normal thyroid areas surrounding cancer cells are also visible. Original magnification x40.

To ascertain the possible role of the components of the AHR pathway in the pathogenesis of PTC, the steady-state levels of *AHR*, *AHRR* and *ARNT* mRNAs in tumoral specimens and paired normal tissues were evaluated by quantitative Real-time PCR (qPCR). The mean expression levels of *AHRR* (2.27 ± 1.80 *vs* 1.81 ± 2.06, *p* = 0.065) and *ARNT* (1.68 ± 0.70 *vs* 1.62 ± 0.89, *p* = 0.843) were similar in tumoral and paired normal tissue from 51 PTC, while a significant increase of *AHR* (3.36 ± 2.83 *vs* 2.08 ± 1.84, *p* < 0.0001, Figure 1A) was observed.

PTCs were then grouped according to the BRAF mutational status, being mutated in 49% (25/51) of cases, and possible differences in term of AHR expression were investigated. As shown in Figure 1 (Panels B and C), significant differences between tumoral and normal tissues could be observed in the BRAF mutated PTCs (mean of differences 1.88, 95% CI: 1.16–2.59, *p* < 0.0001), but not in those carrying the wild-type form (0.69, 95% CI: 0.17–1.55, *p* = 0.20). Moreover, as expected, BRAF^V600E^ cases expressed AHR at a higher level than the BRAF^wt^ (2.27 ± 1.01 *vs* 1.44 ± 1.21, *p* = 0.0003, Figure 1D). Conversely, we could not find any correlation when samples were grouped according to RAS genes mutational status.

Parallel western blot experiments testing AHR expression in normal*/*tumoral match-pair samples showed a good positive correlation between AHR mRNA and protein expression. This confirms that the difference in AHR expression between PTC and its normal paired tissue was significantly higher in tumors carrying the BRAF^V600E^ compared to BRAF^wt^ PTC (Figure 1E).

AHR protein expression was then evaluated in tumoral specimens by immunohistochemistry. As shown in Figure 1F and in accordance with a previous report [14], a weak cytoplasmic and nuclear AHR immunostaining was observed in normal thyroid areas surrounding the neoplastic tissue. In BRAF^V600E^ PTC cases a strong and homogeneous cytoplasmic AHR staining has been detected. Conversely, BRAF^wt^ PTC samples displayed lower cytoplasmic or absent AHR staining (Figure 1G).

To further validate the AHR expression trend observed in our series, and to establish if this might be associated to an increased AHR signaling, a large-scale meta-analysis of microarray data in the public domain has been performed. Gene expression data from five previously published microarray datasets were retrieved from GEO and integrated with a custom methodology. Patients’ tissue samples included normal thyroid (NT, *n* = 73), follicular thyroid carcinoma (FTC, *n* = 14), poorly differentiated thyroid carcinoma (PDTC, *n* = 4), PTC (*n* = 128) and anaplastic thyroid carcinoma (ATC, *n* = 22). Expression data showed an upregulation of *AHR* in PTC compared to normal thyroid tissues (*p* < 0.0001, Supplemental Figure 1A), while *ARNT* did not show significant differences between the two groups. We then evaluated the expression of phase I (e.g. CYP1A1 and CYP1B1) and phase II (e.g. NQO1) biotransforming enzymes. A four-fold increase of *CYP1B1* was observed in PTC compared to NT (Supplemental Figure 1B), while only a slight 1.5-fold increase was observed for *NQO1* (*p* < 0.0001) (Supplemental Figure 1C). In contrast, *CYP1A1* did not significantly change between the two groups (Supplemental Figure 1D). Clinical meta data associated to expression profiles were further analyzed to assess if *AHR* transcription expression may be a function of thyroid disease progression or histotype. In spite of the different groups’ size and the lack of a statistical significance, in FTC, *AHR* expression was lower than NT, while ATC showed expression levels higher than NT but similar to PTC (Supplemental Figure 2).

To correlate clinical phenotype with AHR expression, the 51 PTC patients in our series were divided into 2 groups (AHR high- or low-expressing PTCs) according to the receptor median expression values. The clinico-pathological features of the 51 PTC patients were summarized in supplemental Table 1. At the end of a median follow-up of 65 months, 80% of patients were cured, 16% showed a persistent disease, while 4% deceased during the follow-up. Although PTC patients with high AHR expression levels showed preferentially low (I and II) than high (III and IV) PTC stages (68% vs 32%) – low AHR expressing cases are instead equally shared between the two groups – no significant correlation between AHR expression and patients’ age, tumor size, lymph node and distant metastases, and poor outcome could be established.

### AHR expression in thyroid cell lines and effect of *BRAF*^V600E^ on AHR expression

**Figure 2:**
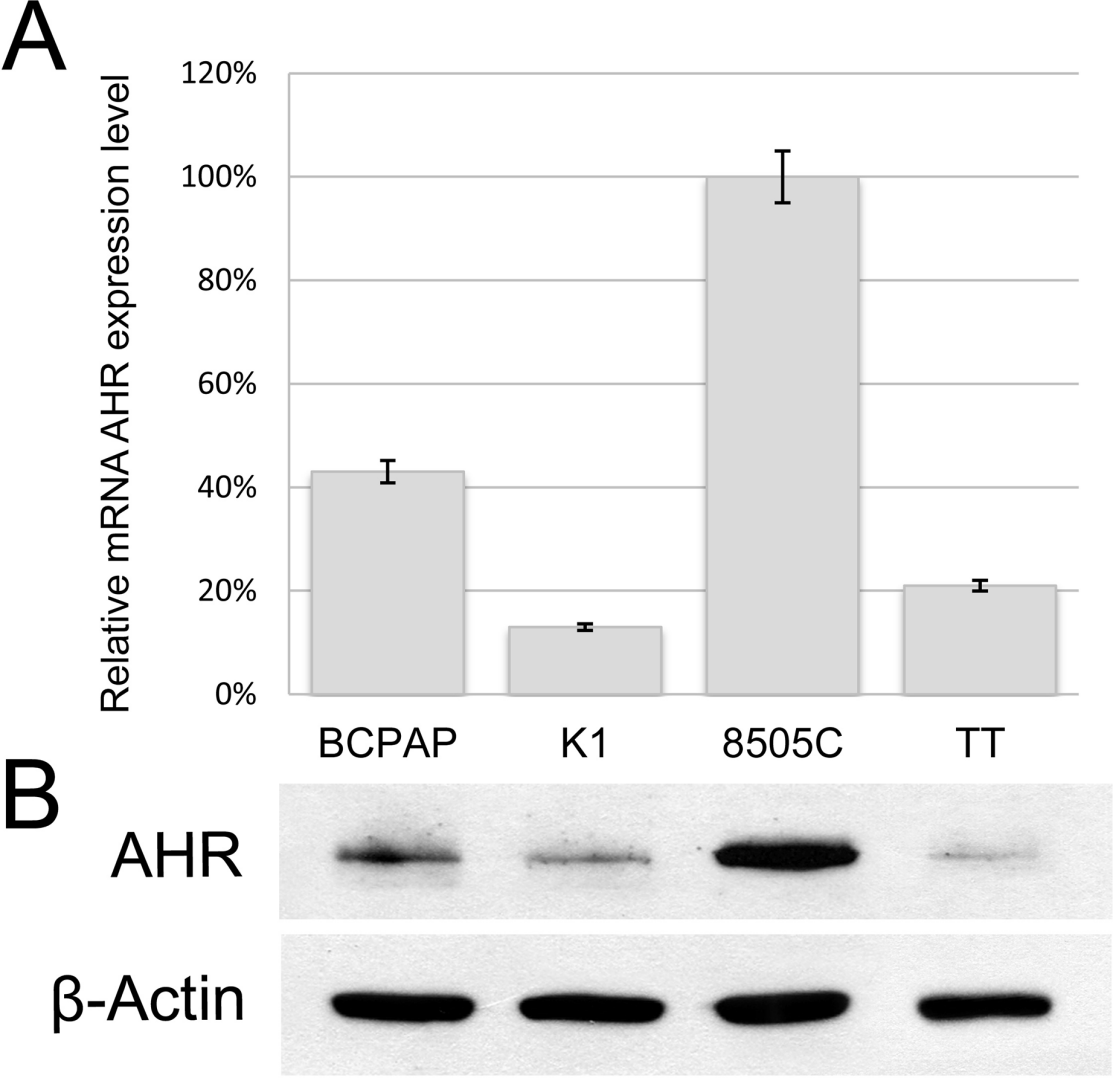
AHR expression in thyroid tumors derived cell lines The PTC-derived BCPAP and K1, the medullary thyroid cancer cell line TT, and the 8505C established from an undifferentiated thyroid carcinoma were evaluated for AHR expression at both mRNA **A.** and protein level **B.** Samples were corrected for protein loading by β-Actin. Error bars represent standard deviations.

AHR expression levels were then assessed in four thyroid cell lines, all but one (TT) carrying the BRAF^V600E^ in either a homozygous (BCPAP) or heterozygous state (K1 and 8505C). As shown in Figure 2, 8505C showed the highest expression both at mRNA and at protein level. AHR was instead detected at lower but similar protein levels in BCPAP and K1, while apparently no expression was found in TT cell line.

**Figure 3:**
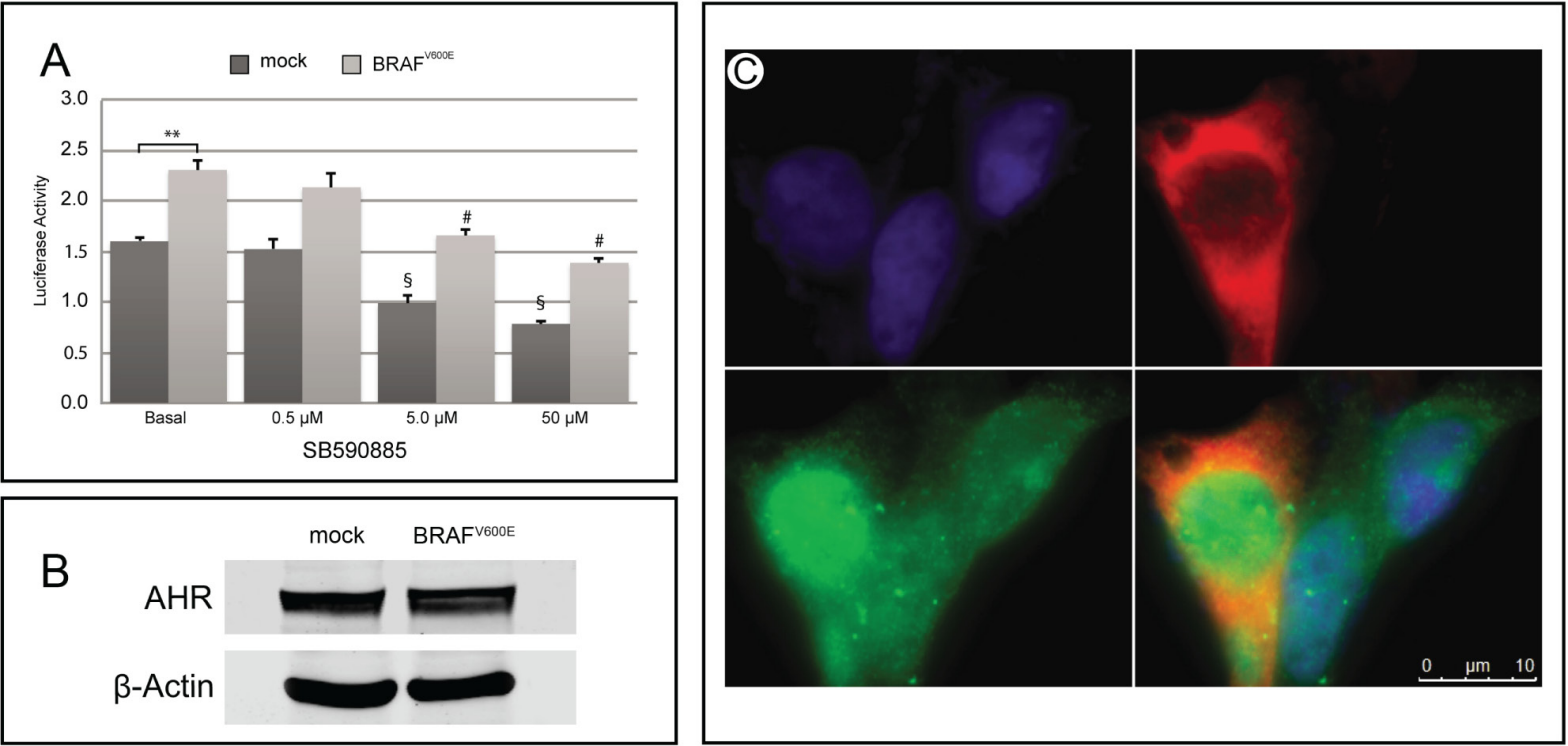
Effect of BRAF^V600E^ on AHR expression/activity in HEK293 cells **A.** HEK293 cells were co-transfected with the XRE-luc reporter plasmid and either the human Flag-tagged BRAF^V600E^ plasmid or the empty vector pcDNA3.1 and treated with increasing concentration of the BRAF inhibitor SB590885. The relative activity was adjusted for transfection efficiency using pRL-TK. Error bars represent standard deviation. ** reflects *p* < 0.01 value for luciferase activity in mock versus BRAF^V600E^ transfected cells at basal level; # § reflect *p* < 0.01 values for treated versus untreated cells. **B.** Representative Western blot analysis of AHR in HEK293 cells transfected with either BRAF^V600E^ or pcDNA3.1. β-Actin was used to normalize for loading variations. **C.** Immunofluorescence staining of Hoechst (blue), Flag (red) and AHR (green) in BRAF^V600E^ transfected HEK293. Only one of three cells in the picture is positive for Flag-tagged BRAF^V600E^, for which an increased in nuclear AHR could be observed. All experiments were performed at least in triplicate.

To exclude that the association between BRAF^V600E^ and AHR overexpression was fortuitous and to ascertain if an active BRAF protein up-regulates AHR, the BRAF^wt^ HEK293 cells, were co-transfected with a human BRAF^V600E^ and a reporter plasmid in which luciferase expression is driven by AHR, through XRE elements. The constitutively active BRAF^V600E^ induces a significant increase in luciferase activity compared to mock transfected cells (44% ± 13%, *p* < 0.01, Figure 3A). Interestingly, this positive stimulus could be reversed by the kinase inhibitor SB590885 acting on both the exogenous BRAF^V600E^ and/or the endogenous BRAF, in a dose dependent manner (Figure 3A). To establish the reason for such increase, quantitative and qualitative evaluations of BRAF^V600E^ effect on AHR expression and/or localization were performed. BRAF^V600E^ apparently does not induce AHR expression (Figure 3B), but rather a tendency to increase nuclear translocation (Figure 3C).

**Figure 4:**
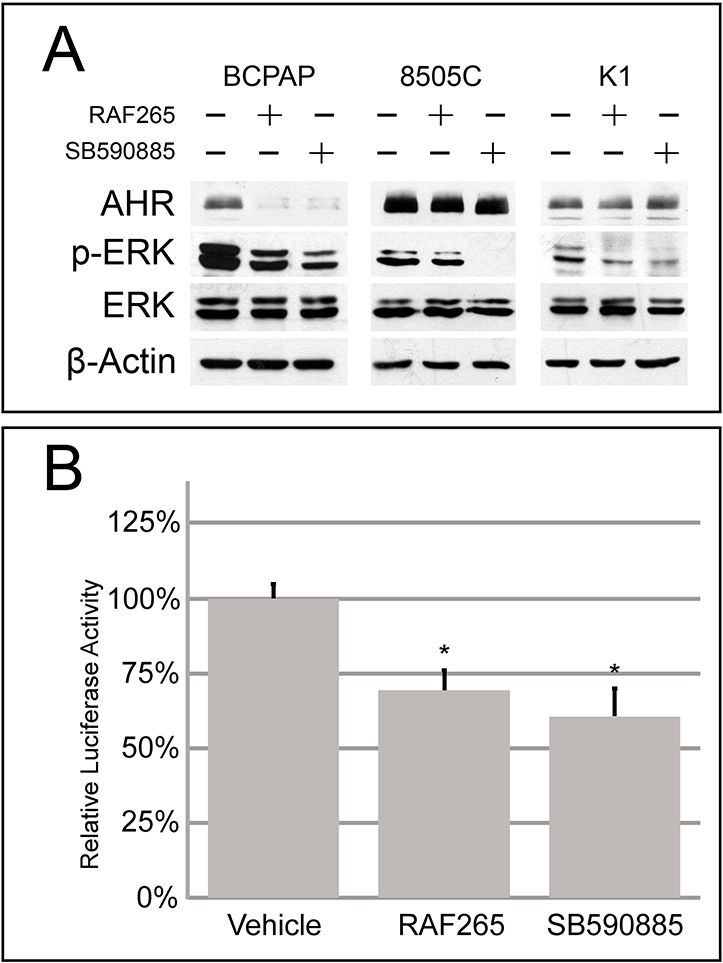
Effect of BRAF inhibitors on AHR expression and activity in thyroid cell lines **A.** Representative Western blot analysis of BCPAP, 8505C and K1 cells treated with IC50 doses of RAF265 and SB590885 for the expression of AHR, phospho-ERK and total ERK. Samples were corrected for protein loading by β-Actin. **B.** Relative luciferase activity of BCPAP cells transfected with XRE-luc and treated with RAF265 and SB590885 with the same concentration as above. Error bars represent standard deviation *p < 0.05, compared with a group given no drug treatment (one-way ANOVA). All experiments were performed at least in triplicate.

To further study the effect of BRAF inhibitors on AHR expression in thyroid-originating tumor cells, the BRAF^V600E^ carrying cell lines were treated with RAF265 or SB590885, which exert their inhibitory activity with more potency towards BRAF active conformation than the inactive one [4]. As expected *p*-ERK was strongly reduced in all cell lines after both treatments. In the BCPAP the inhibited BRAF activity is followed by a significant decrease of AHR expression (Figure 4A) and activity (Figure 4B), while no effect could instead be observed in 8505C and K1 (Figure 4A).

## DISCUSSION

AHR has been considered for years as a major regulator of xenobiotic-induced carcinogenesis. Now it is becoming widely recognized that AHR and its abnormal expression play an important role in multiple stages of tumor development and progression. Several recently published works, demonstrated that even in the absence of exogenous ligands, AHR is overexpressed and constitutively active in a variety of human and/or rodent tumors including breast cancer, lung adenocarcinoma, pancreatic and prostate cancer [12, 16, 17].

In the present work, we clearly demonstrated in a large series of PTC – and further confirmed by data emerging from a large-scale meta-analysis of microarray studies in thyroid tumors – an increased expression/activity of AHR, independently from its nuclear dimerization partner ARNT and its repressor AHRR, but strictly related to a constitutive active MAPK/ERK pathway.

A strong body of evidences supports the mutual interaction between the AHR and MAPK/ERK pathways [15, 18]. Our finding that BRAF^V600E^ mutation modulates AHR levels, localization and activity in PTC in an exogenous ligand-independent manner further reinforces this concept. Previous reports demonstrated that typical AHR activators (e.g. TCDD, benzo[a]pyrene) trigger MAP kinases at different levels and in different cellular systems [15, 18]. On the other hand, MAPK are key players in regulating AHR function and stability [19–21]. Changes in the AHR levels and the enhanced TCDD-initiated transactivation potential of the receptor have been indeed observed in cell overexpressing constitutively active ERK1 or MEK1 [20, 22]. Moreover, AHR is significantly expressed in a large subset of N-RAS mutated cell lines whose sensitivity to the MEK inhibitor PD0325901 positively correlates with the receptor expression [13]. The direct or indirect involvement of serine/threonine kinases in AHR function regulation is further strengthened by the observation that phosphorylation of AHR co-chaperons HSP90 and of AHR-associated ancillary proteins, including ARNT, modulates the formation of a functional cytosolic AHR multicomponent complex [23] and/or potentiates the transcriptional activity of AHR/ARNT complexes [20].

In most tumoral specimens in our series, AHR was abundantly expressed in the cytoplasm of papillary thyroid cancer cells and was instead absent in the normal adjacent tissue. Although our data do not permit to distinguish whether the BRAF^V600E^ either induces an increased expression/stability or reduces AHR degradation, by analogy with ERK phosphorylation, we suggest that BRAF^V600E^ might stabilize the AHR, increase its nuclear uptake and protect it from proteasome digestion [24]. As expected, cytosolic AHR overexpression may be associated to the consequent enhancement of AHR-regulated downstream gene expression induction [25]. Accordingly, by a large-scale meta-analysis of published microarray data we observed an increased phase I (*CYP1B1*) and phase II (*NQO1*) biotransforming enzymes, supporting the idea that in PTC cells AHR can potentially heterodimerize with ARNT in the absence of ligands.

Three different aspects emerging from our cellular studies further support the inductive role of BRAF^V600E^ on AHR expression/activity. 1- AHR is expressed in 8505C, BCPAP and at lower level in K1 all of them carrying the BRAF^V600E^, while it is barely detectable in the BRAF^wt^ TT cells. Moreover, in HEK293 with a constitutively active MAPK pathway, the AHR activity was significantly increased. 2- The positive stimulus of BRAF^V600E^ on AHR can be significantly reverted by kinases inhibitors in both BCPAP and HEK293. By specifically blocking BRAF, RAF265 and SB590885 therefore not only reduce cell proliferation and promote apoptosis in BCPAP as we previously demonstrated [4], but also inhibit AHR expression/activity pathway. Additional data could be provided by the use of selective MEK inhibitors. However, the possible concomitant effects of these drugs on AHR [26, 27], prevents their use for studying the effects of blocking the MAPK/ERK pathway downstream of BRAF on AHR activity. In K1 and 8505C cell lines, however, the inhibition of ERK phosphorylation is not accompanied by a decrease in AHR expression. The reason of this uncoupling is unclear, however it is reasonable to hypothesize that other molecular pathways may sustain AHR expression. Further studies would help clarifying this point. 3- The introduction of a constitutive active BRAF^V600E^ in HEK293 induces AHR activity. This effect seems not to reflect an increase in AHR expression as observed in PTC tumoral specimens, but more likely an increased nuclear shuttling. This may reflects the tissue-specific regulation of AHR localization [28].

Both AHR and CYP1B1 represent potential targets for chemoprevention in several cases. Some AHR agonists inhibit the growth of pancreatic cancer cells expressing AHR at high levels [29]. Many flavonoids may prevent or improve tumorigenic outcomes by reducing AHR and/or CYP1A1/CYP1B1 activity, or by preventing PAH-induced genotoxicity [30]. CYP1B1 represents a therapeutic target also for potential anticancer drugs that can be metabolically activated by this biotransforming enzyme [29]. For instance 3,4-Methylenedioxy-3′,4′,5′-trimethoxy chalcone, has shown promise in the treatment and prevention of gastrointestinal tumors in mouse models [31]. Our data raise hence the possibility to consider AHR and CYP1B as possible novel therapeutic targets also for PTC. Anyway, the lack of any link between AHR expression and poor prognostic factors in our PTC series, together with the lack of any correlation with more de-differentiated histotypes from meta data analysis, prompt us to consider AHR as one of initial mediators of thyroid cancer development endorsed by BRAF, rather than a driver of the disease progression process.

In conclusion, this study supports end extends our previous findings concerning the relationship between BRAF^V600E^ and AHR expression in human PTC [14]. Indeed, it provides the first evidence that BRAF^V600E^ activating the MAPK/ERK signaling is able to upregulate the AHR pathway in these tumors, suggesting that targeting the AHR pathway might have potential therapeutic benefit in their clinical management. It raises, however also novel relevant issues we aim to clarify in the next future, including the role of the proteasome in the high AHR expression in mutated PTCs and the nature of the interaction between BRAF and AHR (i.e. direct or mediated through other proteins). Finally, as BRAF somatic mutations characterize other human malignancies including colon cancer and melanoma, future research should clarify if the BRAF-mediated AHR activation is peculiar of PTC or a more general mechanism.

## MATERIALS AND METHODS

### Patients, DNA extraction and mutation analysis

From 2010 to 2013, we collected a series of paired-normal and PTC tissues from 51 patients: 17 males and 34 females with a mean age 46 years (range 5–69) with a median follow-up of 65 months (range 15–79). All patients were treated with total thyroidectomy for PTC, and therapeutic neck dissection was performed in patients with standard indications. According to the 6th TNM classification 29 were stage I, 2 were stage II, 14 were stage III and 6 were stage IV. Follow-up or survival time was defined as the time from the initial surgical treatment to patient's death due to PTC or to the most recent clinic visit. Patients gave written informed consent for their thyroid tissues to be used for research purposes. The local ethical committee approved the study.

DNA was extracted from frozen tissues after surgery using the DNeasy Blood and Tissue kit (Qiagen, Italy), according to the manufacturer's protocol. Mutation analyses were performed for BRAF (NM_004333.4), N-RAS (NM_002524.3; exons 2 and 3), K-RAS (NM_033360.2; exons 2 and 3), and H-RAS (NM_005343.2; exons 2 and 3) by direct sequencing, as described [14, 32].

### Cell lines and treatments

Four human thyroid cell lines that have been recently authenticated to be unique thyroid cancer cell lines [33, 34], were used: the PTC-derived BCPAP (Leibniz Institute-DSMZ, Germany) and K1, the medullary thyroid cancer cell line TT, and the 8505C established from an undifferentiated thyroid carcinoma (ECACC, Sigma-Aldrich, Italy). In addition, the human embryonic kidney HEK293 cell line (American Type Culture Collection, VA) was used for transfection experiments. All cell lines but HEK293, which was maintained in DMEM, were cultured in RPMI 1640 (Gibco, Italy) supplemented with 10% FBS (Gibco), L-glutamine (2 mM) and penicillin/streptomycin (100 IU/mL/100 μg/mL, respectively). Adherent monolayer cultures were maintained in T75 culture flasks and incubated at 37°C with 5% CO_2_ until they achieved 85% confluency. Cells were detached using 0.25% trypsin (Sigma-Aldrich) and plated into T75 flasks at a density of 2 × 10^6^ cells.

Novartis International (Basel, Switzerland) kindly provided RAF265, while SB590885 were purchased from Selleckchem (Houston, TX), and dissolved in DMSO following the manufacturer's instructions. Cells were incubated with the drugs for 72 hours at the IC50 doses we recently established [4]. IC50 for RAF265 was 0.12 μM, 0.55 μM and 1.32 μM in 8505C, BCPAP and K1 cells, respectively. IC50 for SB590885 was 5.2 μM in BCPAP and K1 cells and 6.2 μM in 8505C cells.

### Cell transfection and dual-luciferase assay

Twenty-four hours before the experiment, BCPAP (2.5 × 10^5^ cells/well) or HEK293 cells (2 × 10^5^ cells/well) were seeded in 12-well plates. Cells were transiently transfected as reported elsewhere [35] using 2 μl of Lipofectamine 2000 (Invitrogen, Italy) and 1.5 μg of total DNA consisting of the Flag-tagged BRAF^V600E^/pcDNA3.1 plasmids, and/or XRE-luc, and pRL-TK (Promega, Italy). After 6 hours incubation the medium was removed, proteins harvested in passive lysis buffer and the relative luciferase activity measured with the Dual Luciferase Reporter Assay System and a GloMax 20/20 luminometer (Promega), according to the manufacturer's instructions.

### Immunofluorescence microscopy

HEK293 cells were seeded in cover-glass-bottom microwell dishes and transfected with either the Flag- tagged BRAF^V600E^ plasmid or the empty vector pcDNA3.1, as described above. After 24 h, cells were washed twice with PBS, fixed in 4% paraformaldehyde in PBS for 10 min at room temperature, and permeabilized incubating with 0.3% Triton X-100 and BSA 3% in PBS for 30 min. Cells were stained for endogenous AHR and exogenous BRAF, by incubating with a 1:300 rabbit anti-FLAG polyclonal antibody (F7425, Sigma-Aldrich) and a mouse anti-AHR monoclonal antibody (clone 3B12, Novus Biologicals, 1:100) for 1 h at RT. The cells were then washed three times in PBS and incubated with AlexaFluor™ 488 or 594-labeled secondary antibodies (1:250 dilution) (Life Technologies, Italy) for 1 hour at RT. The cells were subsequently washed three times in PBS and nuclei counterstained with 1.5 μg/mL Hoechst 33258 (Sigma-Aldrich) and mounted with Fluorescent Mounting Medium (Dako, Cat.No. S3023).

The preparations were examined with a Leica DMI6000CS fluorescence microscope (Leica Microsystems CMS) using a 100 × /1.40 oil-immersion objective. Images were acquired by means of a DFC365FX camera and analyzed with Leica LAS-AF 3.1.0 software.

### Immunohistochemistry

In a subgroup of nineteen PTC cases (ten BRAF^wt^ and nine carrying the BRAF^V600E^) for which enough material was available, immunohistochemistry was performed on formalin-fixed, paraffin-embedded 4–6 μm thick tissue sections, using a polyclonal rabbit anti-AHR antibody (sc-5579, Santa Cruz Biotechnology, USA, 1:50). Appropriate positive and negative controls were run concurrently. Immunostaining for AHR was semi-quantitatively scored as previously reported [14]. Two different pathologists assessed the analysis in blind. The intensity of the staining was indicated as weak, moderate or strong. The subcellular localization of staining was also considered (cytoplasmic, nuclear or both).

### Meta-analysis of microarray expression data

Raw microarray gene expression profiles were obtained from the NCBI Gene Expression Omnibus (GEO) [36]. We considered five series (GSE53157 [37], GSE33630 [38], GSE29265, GSE3678 and GSE27155 [39]), which comprise 294 samples. Based on tissue type, 241 samples were selected, organized in a proper database, using the open source web application A-MADMAN [40] and considered for further analysis. For details, see Supplemental Materials and Methods.

### Protein extraction and western blotting

Proteins were extracted from the different cell lines (6 × 10^5^ cells into 60 mm cell culture dishes) possibly incubated with RAF265 and SB590885 as described elsewhere [41]. Briefly, cells were PBS-rinsed, lysed in RIPA buffer supplemented with proteases inhibitors and clarified by centrifugation. Available tumoral specimens and normal tissue pairs were re-suspended in lyses buffer and homogenized with Tissue Lyser (Qiagen). Protein concentrations were determined using the Bio-Rad DC protein assay kit (BioRad, Italy) following the manufacturer's instructions. For each sample, 20 μg were resolved by SDS-PAGE on a 10% NuPAGE gel (Invitrogen) and transferred onto nitrocellulose membrane by Trans-Blot Turbo transfer system (BioRad). Membranes were blocked for 2 h with 5% non-fat dry milk, incubated overnight at 4°C with a primary antibody [1:1000 anti-AHR, (clone 3B12, Novus Biological), 1:1000 anti-Erk1/2 and anti-phospho-Erk1/2 (Thr202/Tyr204) (Cell Signaling, Euroclone, Italy)] followed by a 1 h incubation with an HRP-conjugated secondary antibody (Jackson ImmunoResearch Laboratories, US). Expression was corrected for differences in protein loading by probing blots with mouse anti-β-actin antibody (1:5000, clone AC-15, Sigma-Aldrich). Blots were developed using Pierce ECL Substrate and exposed to CL-XPosure Film (Thermo Scientific, Rockford, US). Band intensity was quantified with Image J software 1.44p.

### RNA isolation, reverse transcription and qPCR

Total RNA from frozen tissue after surgery, as well as from each thyroid cell line was extracted using TRIzol (Invitrogen) as previously reported [42]. RNA yield was determined on a spectrophotometer (NanoDrop Technologies, Wilmington, USA). One μg of DNase-treated RNA was reverse-transcribed using M-MuLV Reverse Transcriptase RNase H- (Euroclone) according to the manufacturer's recommendations. qPCR experiments were performed according to the MIQE guidelines [43]. For details, see Supplemental Materials and Methods.

### Statistical analysis

We calculated proportions and rates for categorical variables, means ± standard deviations, or medians and ranges for parametric or non-parametric variables. In qPCR experiments, groups were compared with the Mann-Whitney test or Wilcoxon test for quantitative variables not normally distributed. The MedCalc version 14.7 (MedCalc software, Ostand, Belgium) was used to manage patients’ dataset and for the statistical analyses. The significance level was set at *p* < 0.05 for all tests.

## SUPPLEMENTARY DATA


