# Supplementary material for: A constitutive active MAPK/ERK pathway due to BRAFV600E positively regulates AHR pathway in PTC
Source: Oncotarget. 2015 Sep 16;6(31):32104–14. doi: 10.18632/oncotarget.5194 (PMC4741662; doi:10.18632/oncotarget.5194)
Supplement: Supplementary file 1 [file oncotarget-06-32104-s001.pdf]

SUPPLEMENTARY DATA

Meta-analysis of microarray expression data

A careful quality control check was carried out with the R/Bioconductor package arrayQualityMetrics [1] and samples flagged as outliers according to NUSE and RLE metrics were excluded. Selected samples were obtained from two different generations of Affymetrix Genechip technologies in particular HG-U133A e HG-U133Plus2. Gene expression profiles have been generated from raw CEL files using an ad-hoc normalization step called Virtual-Chip [2, 3] and normalized using RMA [4]. In Virtual-Chip, raw expression data (i.e., CEL files) obtained from at least two different platforms are integrated using an approach inspired by the generation of custom Chip Definition Files, CDFs [5, 6]. In custom CDFs, probes matching the same transcript, but belonging to different probes sets, are aggregated into putative custom-probe sets, each including only those probes with a unique and exclusive correspondence with a single transcript. The probes included in the virtual CDF are those shared among the platforms of interest, with the additional condition of generating custom probe set of at least 4 probes. The virtual CDF can be derived from any custom CDF, e.g., those developed by Dai and publicly accessible at the Molecular and Behavioral Neuroscience Institute Microarray Lab website.

Finally, the virtual CDF can be used as the geometry file in RMA as far as the original CEL files are properly re-mapped to match the topology described in the virtual CDF. Re-mapped CEL files, called virtual CEL file, are homogeneous in terms of platform and gene expression data can be generated with a single step of background correction, normalization and summarization directly from the fluorescence signals of all microarrays composing the meta-dataset.

Differences in means among gene expression levels in different groups of samples were assessed with ANOVA and post-hoc Tukey Honest Significance Differences HSD as implemented in the R statistical environment.

Quantitative real-time PCR (qPCR)

The GoTaq® Probe qPCR Master Mix containing a Carboxy-X-rhodamine passive reference dye (Promega) and TaqMan® Gene Expression Assays for AHR (Hs00169233_m1), AHRR (Hs01005075_m1) and ARNT (Hs01121918_m1) (Life Technologies) were used in an ABI PRISM 7900HT Sequence Detector (Applied Biosystems, Milan, Italy). All samples were tested in duplicate in a MicroAmp 96-well reaction plate sealed with an optical adhesive film (Applied Biosystems) with 20 ng of cDNA template in 20 μl of reaction mixture. No-template controls were included in each run. The qPCR conditions were 95°C for 2 min, followed by 40 cycles at 95°C for 15 s and at 60°C for 1 min. Data were analyzed with the Sequence Detection Software rel. 2.4 (Applied Biosystems), with an automatically-set baseline and a fluorescence threshold adjusted for measuring quantification cycle (Cq) values. A 38-quantification cycle (Cq) limit was set, beyond which the gene was considered undetectable. Validation experiments performed using the standard curve method with five serial dilutions of genomic DNA from control subjects showed identical amplification efficiencies (100% ± 10%) calculated according to: $E = 10^{1/\text{-slope}}\text{-}1$, for all assays. The amount of each target gene relative to the reference gene B-actin (Hs99999903_m1) was ascertained using the ΔΔCq method.

REFERENCES

1. Kauffmann A, Gentleman R, Huber W. arrayQualityMetrics--a bioconductor package for quality assessment of microarray data. Bioinformatics (Oxford, England). 2009; 25:415–416.
2. Fallarino F, Volpi C, Fazio F, Notartomaso S, Vacca C, Busceti C, Bicciato S, Battaglia G, Bruno V, Puccetti P, Fioretti MC, Nicoletti F, Grohmann U, et al. Metabotropic glutamate receptor-4 modulates adaptive immunity and restrains neuroinflammation. Nature medicine. 2010; 16:897–902.
3. Bisognin A, Coppe A, Ferrari F, Risso D, Romualdi C, Bicciato S, Bortoluzzi S. A-MADMAN: annotation-based microarray data meta-analysis tool. BMC bioinformatics. 2009; 10:201.
4. Irizarry RA, Hobbs B, Collin F, Beazer-Barclay YD, Antonellis KJ, Scherf U, Speed TP. Exploration, normalization, and summaries of high density oligonucleotide array probe level data. Biostatistics (Oxford, England). 2003; 4:249–264.
5. Dai M, Wang P, Boyd AD, Kostov G, Athey B, Jones EG, Bunney WE, Myers RM, Speed TP, Akil H, Watson SJ, Meng F. Evolving gene/transcript definitions significantly alter the interpretation of GeneChip data. Nucleic acids research. 2005; 33:e175.
6. Ferrari F, Bortoluzzi S, Coppe A, Sirota A, Safran M, Shmoish M, Ferrari S, Lancet D, Danieli GA, Bicciato S. Novel definition files for human GeneChips based on GeneAnnot. BMC bioinformatics. 2007; 8:446.

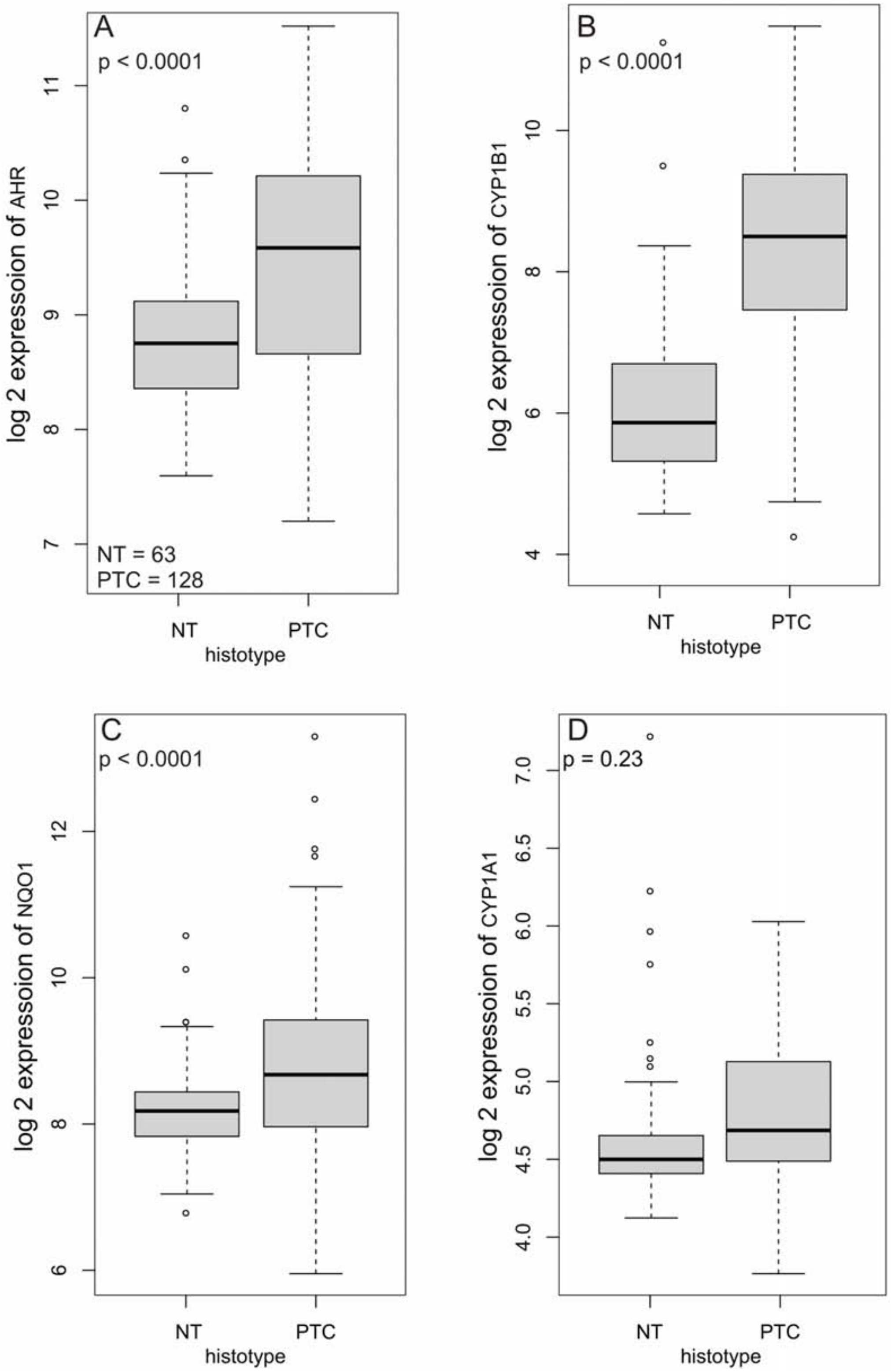

Supplementary Figure S1: PTCs express high levels of genes involved in the AHR pathway compared to normal paired tissues. Box plots of AHR **A.** CYP1B1 **B.** NQO1 **C.** and CYP1A1 **D.** expression in PTC included in the GSE53157, GSE33630, GSE29265, GSE3678 and GSE27155 cohorts. Number of patients and *P*-values from ANOVA tests are displayed.

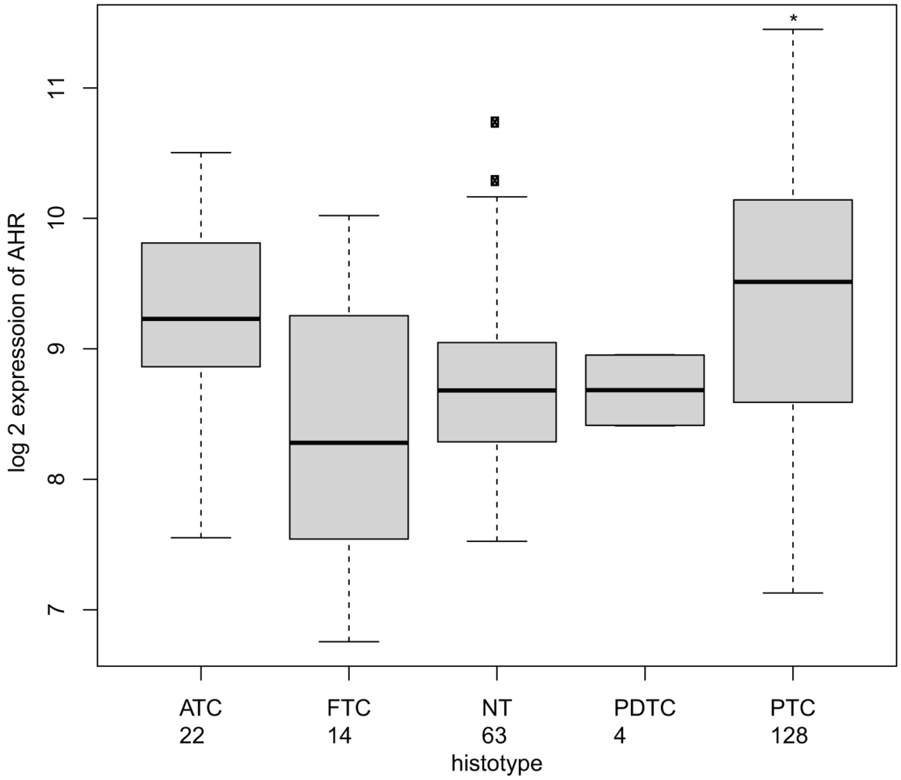

Supplementary Figure S2: AHR expression correlates with PTC. Box plot of AHR expression in Thyroid carcinoma samples classified into four histotypes and compared to normal paired tissues. *P = 0.0004 by analysis of variance (ANOVA). Numbers below the charts represent the amount of samples in each group.

Supplementary Table S1: Association between AHR expression level and different clinicopathological features of PTC patients

Clinicopathological features	No. of cases	AHR expression level		*P*
		High (n, %)	Low (n, %)	
**Age**				
<45	24	13 (54.2)	11 (45.8)	NS
≥45	27	12 (44.4)	15 (55.6)	
**T**				
1 plus 2	18	10 (55.6)	8 (44.4)	NS
3 plus 4	33	15 (45.5)	18 (54.5)	
**Lymph node metastasis**				
No	28	13 (46.4)	15 (53.6)	NS
Yes	23	12 (52.2)	11 (47.8)	
**Stage**				
I	28	16 (57.1)	12 (42.9)	NS
II	2	1 (50.0)	1 (50.0)	
III	13	5 (38.5)	8 (61.5)	
IV	8	3 (37.5)	5 (62.5)	
**Outcome**				
Persistence	8	3 (37.5)	5 (62.5)	NS
Cure	40	21 (52.5)	19 (47.5)	
Death	2	1 (50.0)	1 (50.0)	
